# Early famine exposure and adult disease risk based on a 10-year prospective study of Chinese adults

**DOI:** 10.1136/heartjnl-2019-315750

**Published:** 2019-11-08

**Authors:** Ruogu Meng, Canqing Yu, Yu Guo, Zheng Bian, Jiahui Si, Jia Nie, Ling Yang, Yiping Chen, Huaidong Du, Liyuan Zhou, Yun Liu, Junshi Chen, Zhengming Chen, Liming Li, Jun Lv

**Affiliations:** 1 Center for Data Science in Health and Medicine, Peking University, Beijing, China; 2 Department of Epidemiology and Biostatistics, Peking University Health Science Center, Beijing, China; 3 National Institute of Health Data Science, Peking University, Beijing, China; 4 Chinese Academy of Medical Sciences, Beijing, China; 5 Medical Research Council Population Health Research Unit, University of Oxford, Oxford, UK; 6 Clinical Trial Service Unit & Epidemiological Studies Unit (CTSU), Nuffield Department of Population Health, University of Oxford, Oxford, UK; 7 Liu Zhou Center for Disease Prevention and Control, Liuzhou, China; 8 China National Center for Food Safety Risk Assessment, Beijing, China; 9 Key Laboratory of Molecular Cardiovascular Sciences (Peking University), Ministry of Education, Beijing, China; 10 Institute of Environmental Medicine, Peking University, Beijing, China

**Keywords:** cardiac risk factors and prevention, epidemiology, stroke, chronic coronary disease

## Abstract

**Objective:**

To comprehensively examine the potential impacts of prenatal experience of the Chinese Great Famine on chronic disease risks in the middle age.

**Methods:**

This study included 92 284 participants aged 39–51 years from China Kadoorie Biobank born around the famine period and without major chronic diseases at baseline. We categorised participants into non-famine births (born between 1 October 1956 and 30 September 1958, and 1 October 1962 and 30 September 1964) and famine births (born between 1 October 1959 and 30 September 1961). The outcomes were incident cardiovascular disease, cancer and respiratory system disease. Cox regression was used to estimate adjusted HR and 95% CI for famine exposure. Subgroup analyses were performed according to baseline characteristics.

**Results:**

During a median 10.1 years of follow-up, we identified 4626 incident ischaemic heart disease (IHD) cases, 7332 cerebrovascular disease cases, 3111 cancer cases and 16 081 respiratory system disease cases. In the whole population, prenatal famine exposure was not statistically associated with the risks of developing any chronic diseases in adulthood. However, for urban participants, compared with non-famine births, famine births had a higher risk of cerebrovascular disease (HR 1.18; 95% CI 1.09 to 1.28); such association was not shown for rural participants (p for interaction <0.001). Also, we observed the associations of prenatal famine exposure with IHD (HR 1.15; 95% CI 1.05 to 1.26) and cerebrovascular disease (HR 1.13; 95% CI 1.05 to 1.21) in participants with lower physical activity level, but not in those with higher ones (all p for interaction=0.003).

**Conclusion:**

Our findings indicate that prenatal exposure to the Chinese famine might be associated with an increased cardiovascular risk and such risk may be modified by adult lifestyle.

## Introduction

In addition to established risk factors for non-communicable diseases (NCDs), such as tobacco use, unhealthy diets, reduced physical activity and obesity, growing evidence has suggested that poor prenatal nutrition also has adverse long-term effects on the development of NCDs in later life.^1^ Despite that several studies conducted in developed countries have linked low birth weight and adult-onset diseases, maternal nutrition is only one of the many factors influencing birth weight.^2^ Direct studies regarding prenatal malnutrition and adult-onset diseases were rare due to ethical and practical reasons. Maternal famine experience around the time of pregnancy provides unique opportunities to examine this hypothesis.

The Chinese Great Famine between 1959 and 1961 caused millions of excess deaths.^3^ A few studies in recent years have explored the potential impacts of this famine on adult health, mostly on metabolic disorders and mental illness, and only a few on cancer and respiratory disease.^4–6^ Most of the studies were cross-sectional, in which survival bias is of particular relevance, and the prevalent cases that survive to be in the study may offer a distorted frequency of famine exposure. Also, uncontrolled age differences between pre-famine, famine and post-famine births in some studies have been suggested to explain most effects commonly attributed to the famine.^4^

The China Kadoorie Biobank (CKB) is a large-scale prospective cohort of 0.5 million Chinese aged 30 to 79 years, in which one-fourth of the participants were born during the Chinese Great Famine or 3 years either before or after the famine. In a previous study of this subgroup of CKB participants, we found that prenatal famine experience was associated with an increased risk of adult-onset diabetes.^7^ The present study aimed to further comprehensively examine the potential impacts of famine exposure in early life on major chronic disease endpoints over subsequent 10 years since baseline recruitment in such a group of participants who were born in the 1950s and 1960s and had survived to their midlife.

## Methods

### Participants

As previously described, the CKB cohort enrolled over 0.5 million adults aged 30–79 years from 10 geographically diverse areas across China between 2004 and 2008.^8^ Finally, 512 715 adults had valid baseline data including a completed questionnaire, physical measurements and a written informed consent form, and were followed up thereafter.

We restricted the present study to participants who were born between 1 October 1956 and 30 September 1964 (n=1 20 518). Because of the uncertainty and inconsistency in the exact dates of the start and the end of the Chinese famine across regions, we excluded participants who were born between 1 October 1958 and 30 September 1959 (n=11 577), and between 1 October 1961 and 30 September 1962 (n=14 889).

We further excluded participants with previously diagnosed heart disease (n=944), stroke (n=546) and cancer (n=311). For the analysis of respiratory system disease, we additionally excluded participants who had a self-reported history of emphysema or chronic bronchitis or who was spirometry-measured to have airflow obstruction (AFO) at baseline (n=3552). We excluded prevalent cases at baseline from the analysis to prevent potential survival bias (or incidence/prevalence bias) and the possibility of reverse causation for confounders and effect modifiers. In other words, prevalent cases at baseline were excluded because they over-represent the longer survivors of the diseases. Any risk factors we identify in such a study that includes prevalent cases may be related more to survival with the disease than to the development of the disease. We finally included 92 284 participants for the analysis of cardiovascular disease (CVD) and cancer and 88 808 participants for the analysis of respiratory disease.

### Assessment of famine exposure

We categorised participants into three famine exposure subgroups according to their self-reported birth date at baseline: pre-famine births (born between 1 October 1956 and 30 September 1958), famine births (born between 1 October 1959 and 30 September 1961) and post-famine births (born between 1 October 1962 and 30 September 1964). To balance the differences in age between famine births and other two groups of births, we combined both pre-famine and post-famine births into one group of non-famine births, compared with famine births in the primary analysis.^4^

### Assessment of covariates

Covariate information was obtained from baseline questionnaire including sociodemographic characteristics (sex, region, education and marital status), lifestyle behaviours (tobacco smoking, alcohol consumption, physical activity, and intakes of red meat, fresh fruits and vegetables), women’s reproductive information, and personal and family medical history (hypertension, diabetes, and family history of heart attack, stroke and cancer). The daily level of physical activity was calculated by multiplying the metabolic equivalent tasks (METs) value for a particular type of physical activity by hours spent on that activity per day and summing the MET-hours for all activities. A short qualitative food frequency questionnaire (FFQ) assessed participants’ habitual dietary intake in the past year. Validation study indicated reasonably good validity and reproducibility for FFQ and good reproducibility for physical activity questions.^9 10^ Less than 0.2% of participants were missing for menopausal status and three types of family history. The missing values were categorised into a ‘missing’ group when analysing. No other exposures or covariates contained missing values.

At baseline, trained staff measured body weight, standing height, waist circumference and hip circumference using calibrated instruments.^11^ Body mass index (BMI) was calculated as weight in kilograms divided by height in metres squared. General obesity was determined by the BMI, categorised as underweight and normal weight (<24.0) and overweight and obesity (≥24.0).^12^ Waist:hip ratio (WHR) was calculated as waist circumference divided by hip circumference. Abdominal obesity was determined by the WHR, categorised as <0.90 or ≥0.90 for men, and <0.85 or ≥0.85 for women.^13^ Prevalent hypertension, diabetes and AFO were defined according to measured blood pressure, blood glucose concentration, FEV_1_/FVC, self-reported diagnosis or self-reported medication use at baseline.^11 14^

### Ascertainment of study outcomes

We identified cause-specific morbidity and mortality since the participants’ enrolment into the study at baseline using linkage with local disease and death registries, with the national health insurance system, and by active follow-up. Trained staff, blinded to the baseline information, coded all cases with the 10th revision of the International Classification of Diseases (ICD-10). For the present analysis, primary study outcomes included incident ischaemic heart disease (IHD, I20–I25), major coronary events (including fatal IHD (I20–I25) and non-fatal myocardial infarction (I21–I23)), cerebrovascular disease (I60–I69), ischaemic stroke (I63), haemorrhagic stroke (I61), cancer (C00–C97), respiratory system disease (J00–J99) and chronic obstructive pulmonary disease (COPD, J41–J44).

### Statistical analysis

Participants contributed person-years from the baseline date to the diagnosis of study outcomes, death, loss to follow-up or 31 December 2016, whichever came first. The loss to follow-up in the CKB study refers to a participant whose permanent registered residence has moved out of the jurisdiction of the Regional Coordinating Center. By 31 December 2016, of all 512 715 participants, 44 037 (8.6%) died and 4875 (<1%) were lost to follow-up. The HRs and 95% CIs were estimated by Cox proportional-hazards regression model with age as the underlying time scale, and stratified by study areas.

Multivariable models for associations between early famine exposure and study outcomes were adjusted for sex (for whole cohort only), education, marital status, tobacco smoking, alcohol consumption, physical activity, intakes of fresh fruits, vegetables and red meat, family history of heart attack, stroke or cancer (only adjusted for in corresponding analysis of specific disease category), menopausal status (for women only), BMI, WHR, and prevalent hypertension and diabetes at baseline.

We also examined whether the associations of famine exposure with outcomes differed according to sex (men or women), region (rural or urban), smoking status (current smoker or non-current smoker), alcohol consumption (daily drinker or non-daily drinker), the level of physical activity (<22.7 or ≥22.7 MET-hours/day, according to the median of physical activity level), general and abdominal obesity defined by BMI (<24.0 or ≥24.0 kg/m^2^) and WHR (<0.90 or ≥0.90 for men, and <0.85 or ≥0.85 for women), hypertension (presence or absence) and diabetes (presence or absence) at baseline. The interactions between early famine exposure and these factors were tested by using likelihood ratio test comparing models with and without the cross-product term.

P values are presented as unadjusted for multiple testing unless otherwise indicated. For testing of multiple primary outcomes, a Bonferroni correction was applied to the significance level that divided 0.05 by eight outcomes examined (ie, 0.00625). Statistical analyses were performed using Stata V.14.1 (2013; StataCorp, College Station, Texas, USA). All p values were two sided, and statistical significance was defined as p value <0.05.

## Results

### Baseline characteristics

Of 92 284 participants, pre-famine births (averaged 48.9 years), famine births (46.0 years) and post-famine births (43.0 years) had on average 3 years of age differences (online supplementary table 1). Three famine exposure subgroups showed an increase in the baseline prevalence of hypertension with increasing age. The combination of both pre-famine and post-famine births into one group of non-famine births corrected for the imbalance in age and chronic conditions among subgroups (table 1). However, rural residents were still less common among famine births compared with non-famine births.

10.1136/heartjnl-2019-315750.supp1 Supplementary data

**Table 1:** Baseline characteristics of 92 284 participants according to two famine exposure subgroups

	Non-famine births	Famine births	Difference between groups (95% CI)	P_difference_
No of participants	72 490	19 794	–	
Age at baseline, year	45.7	46.0	−0.4 (−0.4 to −0.3)	<0.001
Men, %	38.9	39.1	0.00 (−0.01 to 0.01)	0.566
Rural area, %	55.6	50.1	0.1 (0.0 to 0.1)	<0.001
Middle school and above, %	68.3	69.6	−0.04 (−0.05 to −0.03)	<0.001
Married, %	95.0	94.8	0.01 (0.00 to 0.01)	0.204
Daily smoking, %	28.5	29.1	−0.00 (−0.01 to 0.00)	0.006
Daily alcohol consumption, %	8.9	9.3	0.000 (−0.005 to 0.004)	0.078
Physical activity, MET-hours/day	25.0	25.2	0.0 (−0.2 to 0.3)	0.125
Average weekly consumption*, day				
Red meat	3.9	3.9	−0.1 (−0.2 to −0.1)	0.879
Fresh vegetables	6.9	6.9	0.02 (0.00 to 0.03)	0.702
Fresh fruits	2.7	2.7	−0.2 (−0.2 to −0.1)	0.936
Family history of, %				
Heart attack	3.9	4.1	−0.01 (−0.01 to 0.00)	0.148
Stroke	19.3	20.3	−0.02 (−0.02 to −0.01)	0.002
Cancer	17.8	18.6	−0.01 (−0.01 to 0.00)	0.013
Postmenopausal women, %	17.9	12.0		<0.001
BMI, kg/m^2^	23.9	24.0	−0.1 (−0.1 to 0.0)	0.001
WHR	0.87	0.88	0.001 (0.000 to 0.002)	<0.001
Hypertension, %	25.1	25.1	0.00 (−0.01 to 0.01)	0.989
Diabetes, %	3.6	4.4	−0.011 (−0.014 to −0.008)	<0.001

The results are presented as adjusted means or percentages, with adjustment for sex and study area, as appropriate.

*Average weekly consumptions of fresh fruits, vegetables and red meat were calculated by assigning participants to the midpoint of their consumption category.

BMI, body mass index; MET, metabolic equivalent of task; WHR, waist:hip ratio.

### Associations of prenatal famine exposure with major chronic diseases

During a median of 10.1 years (0.9 million person-years) of follow-up, we identified 4626 incident IHD cases, 7332 cerebrovascular disease cases, 3111 cancer cases and 16 081 respiratory system disease cases. After adjustment for established and potential risk factors for these chronic diseases, famine exposure in early life was not statistically significantly associated with the risks of developing any chronic diseases of interest in adulthood, although famine birth group tended to have a higher HR for most of the disease outcomes (table 2). The intermediate risk factors, such as overweight/obesity, hypertension and diabetes, did not mediate the association between early famine exposure and adult-onset chronic disease as further adjustment for these factors did not substantially change the effect estimates.

**Table 2:** HRs (95% CIs) for incident chronic disease according to two famine exposure subgroups among 92 284 participants

	Non-famine births	Famine births
Ischaemic heart disease		
Cases (cases/PYs (/1000))	3475 (4.8)	1151 (5.9)
Model 1	1.00	1.06 (0.99 to 1.14)
Model 2	1.00	1.07 (1.00 to 1.15)
Model 3	1.00	1.07 (0.99 to 1.14)
Major coronary events		
Cases (cases/PYs (/1000))	390 (0.5)	125 (0.6)
Model 1	1.00	1.10 (0.89 to 1.36)
Model 2	1.00	1.12 (0.90 to 1.38)
Model 3	1.00	1.11 (0.90 to 1.38)
Cerebrovascular disease		
Cases (cases/PYs (/1000))	5579 (7.8)	1753 (9.0)
Model 1	1.00	1.03 (0.98 to 1.09)
Model 2	1.00	1.04 (0.98 to 1.10)
Model 3	1.00	1.05 (0.99 to 1.11)
Ischaemic stroke		
Cases (cases/PYs (/1000))	3026 (4.2)	980 (5.0)
Model 1	1.00	1.04 (0.97 to 1.12)
Model 2	1.00	1.05 (0.97 to 1.13)
Model 3	1.00	1.06 (0.99 to 1.15)
Haemorrhagic stroke		
Cases (cases/PYs (/1000))	630 (0.9)	186 (0.9)
Model 1	1.00	1.03 (0.87 to 1.22)
Model 2	1.00	1.05 (0.89 to 1.25)
Model 3	1.00	1.06 (0.89 to 1.25)
Cancer		
Cases (cases/PYs (/1000))	2431 (3.3)	680 (3.4)
Model 1	1.00	1.02 (0.93 to 1.11)
Model 2	1.00	1.01 (0.93 to 1.11)
Model 3	1.00	1.01 (0.92 to 1.10)
Respiratory system disease		
Cases (cases/PYs (/1000))	11 815 (18.4)	3390 (19.5)
Model 1	1.00	0.96 (0.93 to 1.00)
Model 2	1.00	0.97 (0.93 to 1.01)
Model 3	1.00	0.97 (0.93 to 1.01)
COPD		
Cases (cases/PYs (/1000))	691 (1.0)	176 (0.9)
Model 1	1.00	1.05 (0.88 to 1.25)
Model 2	1.00	1.06 (0.89 to 1.26)
Model 3	1.00	1.07 (0.90 to 1.27)

Model 1 was adjusted for sex (men or women). Model 2 additionally included education (no formal school, primary school, middle school, high school, college, or university or higher), marital status (married, widowed, divorced or separated, or never married), smoking (never smoker, former smoker who had quit for reasons other than illness, current smoker or former smoker who had quit because of illness: 1–14, 15–24, or ≥25 cigarettes or equivalent tobacco per day), alcohol consumption (non-weekly drinker, former weekly drinker, weekly drinker, daily drinker: <15, 15–29, 30–59 or ≥60 g of pure alcohol), physical activity (MET-hours/day), intakes of fruits, vegetables and red meat (day/week; calculated by assigning participants to the midpoint of their consumption category), family history of heart attack, stroke or cancer (presence or absence; only adjusted for in corresponding analysis of specific diseases) and menopausal status (premenopausal, perimenopausal or postmenopausal; for women only); model 3: additionally included body mass index, WHR, and prevalent hypertension and diabetes at baseline (presence or absence).

COPD, chronic obstructive pulmonary disease; MET, metabolic equivalent of task; PYs, person-years; WHR, waist:hip ratio.

### Sex-specific and region-specific associations

There was no sex heterogeneity in the associations between famine exposure and all study outcomes that met predetermined statistical significance of p value <0.00625 or was clinically meaningful (table 3). For urban participants, early famine exposure was associated with increased risks of cerebrovascular disease (HR 1.18; 95% CI 1.09 to 1.28) and ischaemic stroke (HR 1.18; 95% CI 1.07 to 1.31), which differed from those seen in rural participants (all p for interaction <0.00625) (table 4). The associations of early famine exposure with IHD, cancer and respiratory system disease were consistent between rural and urban areas.

**Table 3:** Sex-specific HRs (95% CIs) for incident chronic disease according to two famine exposure subgroups

	Men (n=35 953)		Women (n=56 331)		P_interaction_
	Non-famine births	Famine births	Non-famine births	Famine births	
No of participants	28 204	7749	44 286	12 045	
Ischaemic heart disease					0.388
Cases	1346	439	2129	712	
Cases/PYs (/1000)	4.8	5.7	4.8	5.9	
HRs (95% CIs)	1.00	1.04 (0.93 to 1.17)	1.00	1.08 (0.99 to 1.18)	
Major coronary events					0.832
Cases	263	85	127	40	
Cases/PYs (/1000)	0.9	1.1	0.3	0.3	
HRs (95% CIs)	1.00	1.09 (0.84 to 1.42)	1.00	1.15 (0.79 to 1.67)	
Cerebrovascular disease					0.016
Cases	2157	640	3422	1113	
Cases/PYs (/1000)	7.8	8.4	7.8	9.4	
HRs (95% CIs)	1.00	1.00 (0.91 to 1.09)	1.00	1.09 (1.01 to 1.17)	
Ischaemic stroke					0.136
Cases	1306	405	1720	575	
Cases/PYs (/1000)	4.6	5.3	3.8	4.8	
HRs (95% CIs)	1.00	1.04 (0.92 to 1.17)	1.00	1.08 (0.98 to 1.20)	
Haemorrhagic stroke					0.271
Cases	314	86	316	100	
Cases/PYs (/1000)	1.1	1.1	0.7	0.8	
HRs (95% CIs)	1.00	0.97 (0.76 to 1.25)	1.00	1.15 (0.91 to 1.45)	
Cancer					0.790
Cases	904	250	1527	430	
Cases/PYs (/1000)	3.2	3.2	3.4	3.5	
HRs (95% CIs)	1.00	1.08 (0.93 to 1.25)	1.00	0.97 (0.87 to 1.09)	
Respiratory system disease					0.063
Cases	4195	1273	7620	2117	
Cases/PYs (/1000)	16.9	18.7	19.5	20.0	
HRs (95% CIs)	1.00	1.00 (0.93 to 1.06)	1.00	0.95 (0.90 to 1.00)	
COPD					0.908
Cases	293	76	398	100	
Cases/PYs (/1000)	1.1	1.0	0.9	0.8	
HRs (95% CIs)	1.00	1.08 (0.83 to 1.41)	1.00	1.06 (0.84 to 1.33)	

Multivariable model was adjusted for the same set of covariates as in model 3 of table 2 except for sex.

COPD, chronic obstructive pulmonary disease; PYs, person-years.

**Table 4:** Region-specific HRs (95% CIs) for incident chronic disease according to two famine exposure subgroups

	Rural areas (n=50 202)		Urban areas (n=42 082)		P_interaction_
	Non-famine births	Famine births	Non-famine births	Famine births	
No of participants	40 288	9914	32 202	9880	
Ischaemic heart disease					0.041
Cases	1754	478	1721	673	
Cases/PYs (/1000)	4.3	4.8	5.4	7.0	
HRs (95% CIs)	1.00	1.07 (0.96 to 1.19)	1.00	1.20 (1.09 to 1.32)	
Major coronary events					0.622
Cases	208	64	182	61	
Cases/PYs (/1000)	0.5	0.6	0.6	0.6	
HRs (95% CIs)	1.00	1.24 (0.92 to 1.66)	1.00	1.08 (0.80 to 1.47)	
Cerebrovascular disease					<0.001
Cases	3235	827	2344	926	
Cases/PYs (/1000)	8.0	8.4	7.4	9.7	
HRs (95% CIs)	1.00	1.04 (0.96 to 1.13)	1.00	1.18 (1.09 to 1.28)	
Ischaemic stroke					0.001
Cases	1531	391	1495	589	
Cases/PYs (/1000)	3.7	3.9	4.7	6.1	
HRs (95% CIs)	1.00	1.04 (0.92 to 1.16)	1.00	1.18 (1.07 to 1.31)	
Haemorrhagic stroke					0.176
Cases	455	118	175	68	
Cases/PYs (/1000)	1.1	1.2	0.5	0.7	
HRs (95% CIs)	1.00	1.00 (0.81 to 1.23)	1.00	1.22 (0.91 to 1.64)	
Cancer					0.361
Cases	1221	315	1210	365	
Cases/PYs (/1000)	3.0	3.1	3.8	3.7	
HRs (95% CIs)	1.00	1.07 (0.94 to 1.22)	1.00	0.97 (0.86 to 1.10)	
Respiratory system disease					0.056
Cases	9764	2764	2051	626	
Cases/PYs (/1000)	29.0	34.5	6.8	6.7	
HRs (95% CIs)	1.00	1.10 (1.06 to 1.15)	1.00	0.95 (0.87 to 1.05)	
COPD					0.149
Cases	595	138	96	38	
Cases/PYs (/1000)	1.5	1.4	0.3	0.4	
HRs (95% CIs)	1.00	0.99 (0.82 to 1.20)	1.00	1.29 (0.87 to 1.93)	

Multivariable model was adjusted for the same set of covariates as in model 3 of table 2.

COPD, chronic obstructive pulmonary disease; PYs, person-years.

### Prenatal famine exposure, lifestyle and health conditions of adults, and major chronic diseases

**Figure 1:**
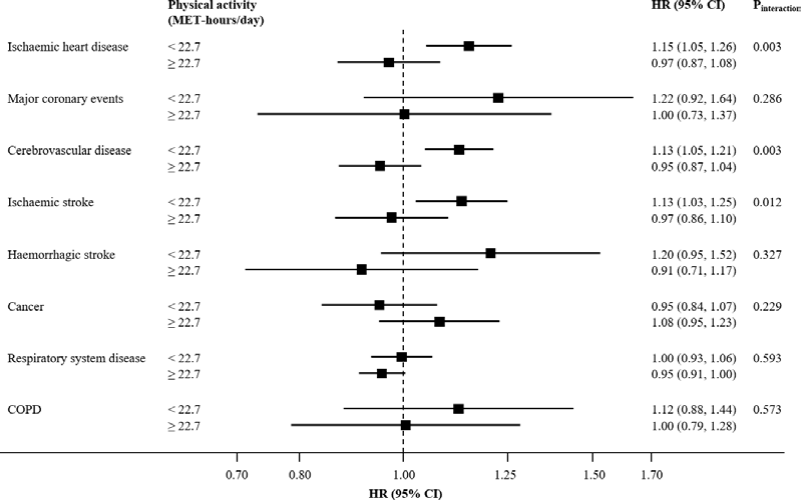
HRs (95% CIs) for association between early famine exposure and incident study outcomes according to physical activity level in adulthood. COPD, chronic obstructive pulmonary disease; MET, metabolic equivalent of task. HRs for incident study outcomes are for comparison between famine births and non-famine births. Squares represent point estimates for HRs, and horizontal lines represent 95% CIs. Multivariable model was adjusted for the same set of covariates as in model 3 of table 2 except for physical activity. A HR greater than 1.0 means an increased risk, and a HR less than 1.0 means a smaller risk.

We also examined whether the associations of early famine exposure with study outcomes differed according to baseline lifestyle factors and chronic conditions. There was no statistically significant heterogeneity for most of the associations across baseline strata (online supplementary tables 2–5). Interestingly, we found that early famine exposure was associated with increased risks of IHD and cerebrovascular disease in participants with lower physical activity level but not in higher ones (p for interaction: 0.003 for IHD and 0.003 for cerebrovascular disease) (figure 1). Compared with non-famine births, the HRs (95% CIs) for famine birth in participants with lower physical activity level were 1.15 (1.05 to 1.26) for IHD and 1.13 (1.05 to 1.21) for cerebrovascular disease; no statistically significant association was seen in those with higher physical activity level.

## Discussion

In this study of over 92 000 middle-aged adults born around the time of the Chinese Great Famine, famine exposure in the fetal stage was not associated with increased risks of major chronic diseases, including CVD, cancer and respiratory system disease, in the subsequent 10 years. However, in the subgroup analysis, we observed an increased risk of cerebrovascular disease associated with prenatal famine exposure in the urban participants, and also increased risks of IHD and cerebrovascular disease in participants with a lower physical activity level.

Two studies performed in the Dutch Famine Birth Cohort have reported that prenatal exposure to the Dutch famine of 1944–1945 was not associated with the subsequent risk of stroke from around 50 to 60 years of age,^15^ but exposure to famine in early gestation was associated with 3-year earlier diagnosis of coronary artery disease.^16^ However, both studies were underpowered with the very small number of cases (less than 20 cases in the exposed group). The Prospect-EPIC Cohort with 7845 middle-aged women also did not find a statistically significant association of exposure to the Dutch famine between ages 0 and 9 years with the subsequent 10-year risks of coronary heart disease and stroke.^17^ Although the present study did not find evidence for the effect of prenatal exposure to famine on the incident risks of both IHD and cerebrovascular disease in the whole population, a 20% increase in the risk of cerebrovascular disease, especially ischaemic stroke, associated with prenatal famine exposure was seen in urban participants. The reason is unclear, possible explanations for the urban and rural difference in such association include the possibly slighter difference in maternal nutrition between famine and non-famine periods in rural than in urban areas, or higher risk of cerebrovascular disease in rural areas leading to little deleterious effect on the relative scale additionally added by prenatal famine exposure.^18^

The findings of the present study indicated that the increased risks of IHD and cerebrovascular disease from prenatal famine exposure were only shown in participants with lower physical activity level but not in higher ones. Cardiovascular benefits from regular physical activity have been well established.^19^ On condition that intrauterine malnutrition predisposes individuals to CVD in adulthood by hormonal changes in cardiovascular control mechanisms, altered myocardial structure and endothelial dysfunction,^20–22^ actively engaging in physical activity might counteract the adverse effects of fetal life on adult cardiovascular health by mediating vascular function and remodelling through shear stress changes.^23 24^ Higher physical activity in rural than in urban participants might also help explain the aforementioned urban and rural difference in the association of prenatal famine exposure with cerebrovascular disease.

Using data from the Dutch Famine Birth Cohort of 475 women, researchers reported that women who had been exposed to famine in utero had a non-significant higher incidence of self-reported breast cancer (10 cases) compared with unexposed women (five cases) (HR 2.6; 95% CI 0.9 to 7.7).^25^ Another study based on the cancer incidence data between 1983 and 2007 from Shanghai Cancer Registry showed that the age-standardised incidence rates of oesophageal, gastric, colorectal and liver cancers were higher in cohorts exposed to the Chinese famine at ages 0–9 years than in a reference cohort conceived after the famine.^6^ In the present study, we found no association between prenatal famine exposure and total adult cancer risk either in the whole population or any predefined subpopulations. Due to the small number of specific types of cancer cases, no further analysis was conducted. This limitation should be acknowledged.

The Prospect-EPIC cohort, the only prospective study of 7841 women which investigated the association of early famine exposure and later risks of hospitalisation for obstructive airways disease, COPD and asthma, found no association of these disease risks with childhood exposure to the Dutch famine (0 to 9 years), in line with our findings.^26^

Studies in experimental rats have suggested that the development of vital organs, such as brain and lung, was relatively protected when the fetus was exposed to restricted nutrition.^27^ In contrast, the heart, kidney and thymus were the category that reduction in organ weights in proportion to body weight; and the pancreas, spleen, muscle and liver were the category that greater reduction in organ weights than body weight. Both the present and our previous study^7^ to some extent were consistent with this hypothesis.

Linkage to disease and death registries and health insurance system in the CKB cohort enables the collections of a range of disease outcomes. We, for the first time, comprehensively examined the long-term health consequences of the Chinese Great Famine. The strengths of our study included a large sample size and a geographically diverse population. The prospective design, with the exclusion of prevalent cases of major chronic diseases at baseline, could prevent survival bias and minimise the possibility of reverse causation for covariates. We also combined pre-famine and post-famine births into non-famine births as the reference to correct for the age imbalance between comparison groups that was commonly seen in published Chinese famine studies.^4^

## Limitations

There were several limitations to our study. The participants of the present study were those who have survived to their adult years and have not developed major chronic diseases at enrolment. The present study only provided evidence for the association between prenatal famine exposure and disease risk during a 10-year period of middle-aged adults. Prenatal and birth cohort studies are the study design that best allows for the investigation of the continuous lifelong health impact of prenatal exposure to adverse factors but needs decades of follow-up. Because of the lack of individual famine exposure information, we assessed exposure according to the birth dates of participants. Misclassification of famine exposure was inevitable, but should be non-differential on subsequent disease status and have biased our results towards the null. We also excluded participants born in 1 year between famine exposure subgroups to minimise potential misclassification, which was consistent with previous studies.^28 29^ Pre-famine births might have been exposed to famine during their childhood. The mothers of the post-famine births might not have recovered from the famine. Both of these is likely to lead to an underestimation of the effect of fetal famine exposure on adult chronic diseases. The lack of information on other exposures and medical conditions in early life, such as maternal smoking, birth weight and family socioeconomic status, and total energy intake at baseline might lead to residual confounding. However, previous studies showed that the association between early famine exposure with later health outcomes were not altered after adjustment for these factors.^16 30^ Lifestyle covariates were self-reported, and misclassification was inevitable. The physical activity questionnaire was adapted from validated questionnaires used in previous studies in both high-income and Chinese populations, but has not been compared directly with a reference method.

## Conclusions

In conclusion, we found that urban participants had an increased risk of cerebrovascular disease associated with their prenatal exposure to the Chinese Great Famine, and physically inactive participants had increased CVD risk associated with prenatal famine exposure. However, no association was identified between prenatal famine exposure and adult risks of cancer and respiratory system disease. Hunger and food insecurity are still evident in many countries, leading to different manifestations of malnutrition including low birth weight, childhood stunting, anaemia in women of reproductive age and obesity in adults. Our findings offer evidence for an association between nutrition in pregnancy and adult CVD risk and potential modification by adult lifestyle. It is meaningful to encourage CVD prevention throughout the lifespan, from even before conception to later life. Further investigations are warranted to validate our findings.

**Key messages**

**What is already known on this subject?**

- Previous studies have suggested that prenatal malnutrition due to the Chinese Great Famine has profound adverse effects on later health conditions, such as diabetes, metabolic syndrome, hypertension and obesity.
- Reliable evidence about the impact of the Chinese famine on the development of cardiovascular disease (CVD), cancer and respiratory disease in adulthood was limited due to age-related bias and survival bias.

**What might this study add?**

- For adults born around the time of the Chinese Great Famine, famine exposure in the fetal stage was not associated with increased risks of cancer and respiratory system disease in the subsequent 10 years.
- Urban participants had an increased risk of cerebrovascular disease associated with their prenatal exposure to the Chinese Famine.
- Physical inactivity in adulthood exacerbated the adverse effect of prenatal famine exposure on risks of CVD.

**How might this impact on clinical practice?**

- This study provides evidence for encouraging CVD prevention across the whole lifecourse, from nutrition in pregnancy to lifestyle in adulthood.
