# Supplementary material for: Early famine exposure and adult disease risk based on a 10-year prospective study of Chinese adults
Source: Heart. 2019 Nov 8;106(3):213–20. doi: 10.1136/heartjnl-2019-315750 (PMC6968949; doi:10.1136/heartjnl-2019-315750)
Supplement: Supplementary data [file heartjnl-2019-315750supp001.pdf]

Members of the China Kadoorie Biobank collaborative group

International Steering Committee: Junshi Chen, Zhengming Chen (PI), Robert Clarke, Rory Collins, Yu Guo, Liming Li (PI), Jun Lv, Richard Peto, Robin Walters. International Co-ordinating Centre, Oxford: Daniel Avery, Ruth Boxall, Derrick Bennett, Yumei Chang, Yiping Chen, Zhengming Chen, Robert Clarke, Huaidong Du, Simon Gilbert, Alex Hacker, Mike Hill, Michael Holmes, Andri Iona, Christiana Kartsonaki, Rene Kerosi, Ling Kong, Om Kurmi, Garry Lancaster, Sarah Lewington, Kuang Lin, John McDonnell, Iona Millwood, Qunhua Nie, Jayakrishnan Radhakrishnan, Paul Ryder, Sam Sansome, Dan Schmidt, Paul Sherliker, Rajani Sohoni, Becky Stevens, Iain Turnbull, Robin Walters, Jenny Wang, Lin Wang, Neil Wright, Ling Yang, Xiaoming Yang. National Co-ordinating Centre, Beijing: Zheng Bian, Yu Guo, Xiao Han, Can Hou, Jun Lv, Pei Pei, Chao Liu, Yunlong Tan, Canqing Yu. 10 Regional Co-ordinating Centres: Qingdao CDC: Zengchang Pang, Ruqin Gao, Shanpeng Li, Shaojie Wang, Yongmei Liu, Ranran Du, Yajing Zang, Liang Cheng, Xiaocao Tian, Hua Zhang, Yaoming Zhai, Feng Ning, Xiaohui Sun, Feifei Li. Licang CDC: Silu Lv, Junzheng Wang, Wei Hou. Heilongjiang Provincial CDC: Mingyuan Zeng, Ge Jiang, Xue Zhou. Nangang CDC: Liqiu Yang, Hui He, Bo Yu, Yanjie Li, Qinai Xu,Quan Kang, Ziyan Guo. Hainan Provincial CDC: Dan Wang, Ximin Hu, Jinyan Chen, Yan Fu, Zhenwang Fu, Xiaohuan Wang. Meilan CDC: Min Weng, Zhendong Guo, Shukuan Wu,Yilei Li, Huimei Li, Zhifang Fu. Jiangsu Provincial CDC: Ming Wu, Yonglin Zhou, Jinyi Zhou, Ran Tao, Jie Yang, Jian Su. Suzhou CDC: Fang liu, Jun Zhang, Yihe Hu, Yan Lu, , Liangcai Ma, Aiyu Tang, Shuo Zhang, Jianrong Jin, Jingchao Liu. Guangxi Provincial CDC: Zhenzhu Tang, Naying Chen, Ying Huang. Liuzhou CDC: Mingqiang Li, Jinhuai Meng, Rong Pan, Qilian Jiang, Jian Lan,Yun Liu, Liuping Wei, Liyuan Zhou, Ningyu Chen Ping Wang, Fanwen Meng, Yulu Qin,, Sisi Wang. Sichuan Provincial CDC: Xianping Wu, Ningmei Zhang, Xiaofang Chen,Weiwei Zhou. Pengzhou CDC: Guojin Luo, Jianguo Li, Xiaofang Chen, Xunfu Zhong, Jiaqiu Liu, Qiang Sun. Gansu Provincial CDC: Pengfei Ge, Xiaolan Ren, Caixia Dong. Maiji CDC: Hui Zhang, Enke Mao, Xiaoping Wang, Tao Wang, Xi zhang. Henan Provincial CDC: Ding

Meng R, *et al. Heart* 2020; 106:213–220. doi: 10.1136/heartjnl-2019-315750

Zhang, Gang Zhou, Shixian Feng, Liang Chang, Lei Fan. Huixian CDC: Yulian Gao, Tianyou He, Huarong Sun, Pan He, Chen Hu, Xukui Zhang, Huifang Wu, Pan He. Zhejiang Provincial CDC: Min Yu, Ruying Hu, Hao Wang. Tongxiang CDC: Yijian Qian, Chunmei Wang, Kaixu Xie, Lingli Chen, Yidan Zhang, Dongxia Pan, Qijun Gu. Hunan Provincial CDC: Yuelong Huang, Biyun Chen, Li Yin, Huilin Liu, Zhongxi Fu, Qiaohua Xu. Liuyang CDC: Xin Xu, Hao Zhang, Huajun Long, Xianzhi Li, Libo Zhang, Zhe Qiu.

Figure Legends

Supplementary figure 1. Assessment of famine exposure

The upper and lower time axes show the definitions of the Chinese Great famine and famine exposure subgroups (based on birth), separately. Lines between two time axes present a nine-month gestation period.

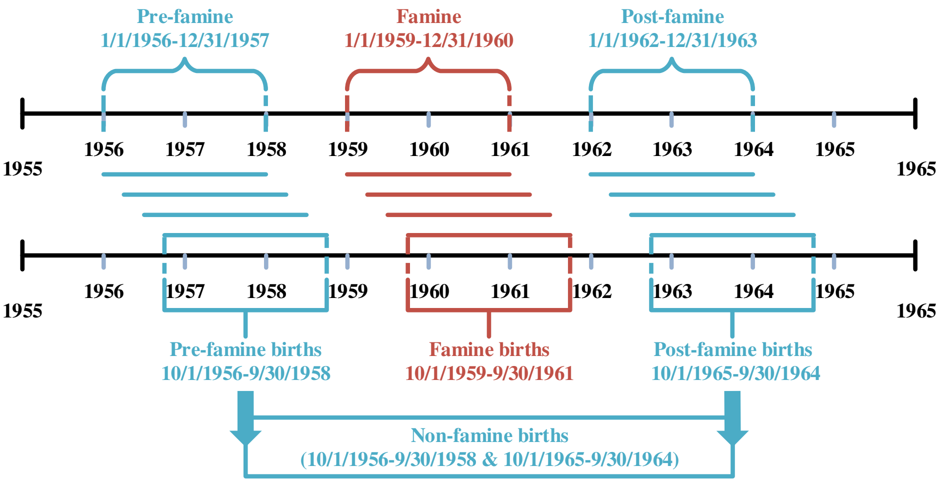

Meng R, *et al. Heart* 2020; 106:213–220. doi: 10.1136/heartjnl-2019-315750

Supplementary table 1. Baseline characteristics of 92,284 participants according to three famine exposure subgroups

	Post-famine births	Famine births	Pre-famine births
No. of participants	39,698	19,794	32,792
Age at baseline, year	43.0	46.0	48.9
Men, %	37.8	39.1	40.2
Rural area, %	56.5	50.1	54.4
Middle school and above, %	77.8	69.6	56.5
Married, %	95.4	94.8	94.5
Daily smoking, %	27.6	29.1	29.4
Daily alcohol consumption, %	8.0	9.3	10.0
Physical activity, MET-hour/day	25.9	25.2	23.9
Average weekly consumption*, day			
Red meat	4.0	3.9	3.8
Fresh vegetables	6.9	6.9	6.9
Fresh fruits	2.8	2.7	2.6
Family history of, %			
Heart attack	3.8	4.1	4.1
Stroke	18.3	20.3	20.6
Cancer	16.9	18.6	18.9
Postmenopausal women, %	4.4	12.1	35.1
BMI, kg/m^2	23.8	24.0	24.0
WHR	0.87	0.88	0.88
Hypertension, %	21.1	25.1	29.9
Diabetes, %	2.9	4.4	4.4

Abbreviations: MET indicates metabolic equivalent of task; BMI, body mass index; WHR,

Meng R, *et al. Heart* 2020; 106:213–220. doi: 10.1136/heartjnl-2019-315750

waist-to-hip ratio.

The results are presented as adjusted means or percentages, with adjustment for sex and study area, as appropriate.

*Average weekly consumptions of fresh fruits, vegetables, and red meat were calculated by assigning participants to the midpoint of their consumption category.

Meng R, *et al. Heart* 2020; 106:213–220. doi: 10.1136/heartjnl-2019-315750

Supplementary table 2. HRs (95% CIs) for association between early famine exposure and ischemic heart disease according to potential baseline risk factors

	**Non-famine births**		**Famine births**		$\mathbf{P_{interaction}}$
	**Cases**	**HR**	**Cases**	**HR (95% CIs)**	
**Ischemic heart disease**					
**Daily smoking**					0.953
No	2,430	1.00	794	1.07 (0.98, 1.16)	
Yes	1,045	1.00	357	1.07 (0.94, 1.21)	
**Daily alcohol consumption**					0.843
No	3,196	1.00	1,054	1.07 (0.99, 1.15)	
Yes	279	1.00	97	1.05 (0.82, 1.34)	
**Physical activity, MET-hour/day**					0.003
<22.7	1,978	1.00	682	1.15 (1.05, 1.26)	
≥22.7	1,497	1.00	469	0.97 (0.87, 1.08)	
**BMI, kg/m^2**					0.623
<24.0	1,538	1.00	500	1.09 (0.98, 1.21)	
≥24.0	1,937	1.00	651	1.05 (0.96, 1.15)	
**WHR**					0.148
Men <0.90, women <0.85	1,430	1.00	472	1.01 (0.91, 1.13)	
Men ≥0.90, women ≥0.85	2,045	1.00	679	1.10 (1.01, 1.21)	
**Hypertension**					0.509
No	2,214	1.00	744	1.04 (0.96, 1.14)	
Yes	1,261	1.00	407	1.10 (0.98, 1.24)	
**Diabetes**					0.459
No	3,239	1.00	1,065	1.07 (1.00, 1.15)	
Yes	236	1.00	86	1.05 (0.80, 1.36)	

Meng R, *et al. Heart* 2020; 106:213–220. doi: 10.1136/heartjnl-2019-315750

	Non-famine births		Famine births		$P_{interaction}$
	**Cases**	**HR**	**Cases**	**HR (95% CIs)**	
**Major coronary events**					
**Daily smoking**					0.966
No	165	1.00	51	1.09 (0.79, 1.52)	
Yes	225	1.00	74	1.12 (0.85, 1.48)	
**Daily alcohol consumption**					0.205
No	337	1.00	102	1.06 (0.84, 1.34)	
Yes	53	1.00	23	1.41 (0.84, 2.38)	
**Physical activity, MET-hour/day**					0.286
<22.7	198	1.00	69	1.22 (0.92, 1.64)	
≥22.7	192	1.00	56	1.00 (0.73, 1.37)	
**BMI, kg/m^2**					0.973
<24.0	178	1.00	54	1.13 (0.82, 1.56)	
≥24.0	212	1.00	71	1.11 (0.83, 1.47)	
**WHR**					0.046
Men <0.90, women <0.85	143	1.00	32	0.74 (0.50, 1.11)	
Men ≥0.90, women ≥0.85	247	1.00	93	1.32 (1.03, 1.70)	
**Hypertension**					0.870
No	197	1.00	65	1.10 (0.82, 1.49)	
Yes	193	1.00	60	1.11 (0.82, 1.50)	
**Diabetes**					1.000
No	352	1.00	110	1.12 (0.89, 1.40)	
Yes	38	1.00	15	1.09 (0.57, 2.08)	

Abbreviations: HR indicates hazard ratio; CI, confidence interval; and MET, metabolic equivalent of task, BMI, body mass index; WHR, waist-to-hip ratio.

Multivariable model was adjusted for sex (men or women), education (no formal school, primary school, middle school, high school, college, or university or higher), marital status (married, widowed, divorced or separated, or never married), smoking (never smoker, former smoker who had quit for reasons other than illness, current smoker or former smoker who had quit because of illness: 1-14, 15-24, or ≥25 cigarettes or equivalent tobacco per day), alcohol consumption (non-weekly drinker, former weekly drinker, weekly drinker, daily drinker: <15, 15-29, 30-59, or ≥60 grams of pure alcohol), physical activity (MET-hour/day), intakes of fruits, vegetables, and red meat (day/week; calculated by assigning participants to the midpoint of their consumption category), family history of heart attack, stroke, or cancer (presence or absence; only adjusted for in corresponding analysis of specific diseases), menopausal status (premenopausal, perimenopausal, or postmenopausal; for women only), BMI, WHR, and prevalent hypertension and diabetes at baseline (presence or absence), as appropriate.

Meng R, *et al. Heart* 2020; 106:213–220. doi: 10.1136/heartjnl-2019-315750

Supplementary table 3. HRs (95% CIs) for association between early famine exposure and cerebrovascular disease according to potential baseline risk factors

	Non-famine births		Famine births		$P_{interaction}$
	**Cases**	**HR**	**Cases**	**HR (95% CIs)**	
**Cerebrovascular disease**					
**Daily smoking**					0.077
No	3,929	1.00	1,256	1.07 (1.00, 1.14)	
Yes	1,650	1.00	497	1.00 (0.90, 1.12)	
**Daily alcohol consumption**					0.367
No	5,101	1.00	1,608	1.06 (1.00, 1.12)	
Yes	478	1.00	145	0.97 (0.79, 1.18)	
**Physical activity, MET-hour/day**					0.003
<22.7	3,232	1.00	1,048	1.13 (1.05, 1.21)	
≥22.7	2,347	1.00	705	0.95 (0.87, 1.04)	
**BMI, kg/m^2**					0.068
<24.0	2,708	1.00	773	0.99 (0.91, 1.08)	
≥24.0	2,871	1.00	980	1.10 (1.02, 1.19)	
**WHR**					0.124
Men <0.90, women <0.85	2,175	1.00	688	0.99 (0.91, 1.09)	
Men ≥0.90, women ≥0.85	3,404	1.00	1,065	1.09 (1.01, 1.17)	
**Hypertension**					0.728
No	3,238	1.00	1,075	1.04 (0.96, 1.11)	
Yes	2,341	1.00	678	1.05 (0.96, 1.15)	
**Diabetes**					0.757
No	5,187	1.00	1,601	1.05 (0.99, 1.11)	
Yes	392	1.00	152	1.04 (0.85, 1.27)	

Meng R, *et al. Heart* 2020; 106:213–220. doi: 10.1136/heartjnl-2019-315750

	Non-famine births		Famine births		$P_{interaction}$
	Cases	HR	Cases	HR (95% CIs)	
**Ischemic stroke**					
**Daily smoking**					0.070
No	2,007	1.00	670	1.09 (0.99, 1.20)	
Yes	1,019	1.00	310	1.01 (0.89, 1.16)	
**Daily alcohol consumption**					0.152
No	2,713	1.00	889	1.08 (1.00, 1.17)	
Yes	313	1.00	91	0.93 (0.72, 1.19)	
**Physical activity, MET-hour/day**					0.012
<22.7	1,760	1.00	596	1.13 (1.03, 1.25)	
≥22.7	1,266	1.00	384	0.97 (0.86, 1.10)	
**BMI, kg/m^2**					0.057
<24.0	1,330	1.00	381	0.98 (0.87, 1.11)	
≥24.0	1,696	1.00	599	1.12 (1.02, 1.24)	
**WHR**					0.180
Men <0.90, women <0.85	1,119	1.00	365	1.01 (0.89, 1.15)	
Men ≥0.90, women ≥0.85	1,907	1.00	615	1.10 (0.99, 1.21)	
**Hypertension**					0.890
No	1,615	1.00	553	1.04 (0.94, 1.15)	
Yes	1,411	1.00	427	1.08 (0.96, 1.21)	
**Diabetes**					0.289
No	2,764	1.00	869	1.05 (0.97, 1.14)	
Yes	262	1.00	111	1.15 (0.91, 1.46)	
**Haemorrhagic stroke**					
**Daily smoking**					0.542

Meng R, *et al. Heart* 2020; 106:213–220. doi: 10.1136/heartjnl-2019-315750

	Non-famine births		Famine births		$P_{interaction}$
	Cases	HR	Cases	HR (95% CIs)	
No	393	1.00	119	1.10 (0.89, 1.37)	
Yes	237	1.00	67	0.98 (0.74, 1.30)	
**Daily alcohol consumption**					0.695
No	559	1.00	167	1.07 (0.89, 1.28)	
Yes	71	1.00	19	0.99 (0.58, 1.68)	
**Physical activity, MET-hour/day**					0.327
<22.7	326	1.00	100	1.20 (0.95, 1.52)	
≥22.7	304	1.00	86	0.91 (0.71, 1.17)	
**BMI, kg/m^2**					0.594
<24.0	323	1.00	86	1.01 (0.79, 1.29)	
≥24.0	307	1.00	100	1.11 (0.87, 1.40)	
**WHR**					0.758
Men <0.90, women <0.85	229	1.00	66	1.07 (0.80, 1.42)	
Men ≥0.90, women ≥0.85	401	1.00	120	1.05 (0.85, 1.30)	
**Hypertension**					0.728
No	204	1.00	63	1.06 (0.79, 1.43)	
Yes	426	1.00	123	1.05 (0.85, 1.29)	
**Diabetes**					0.723
No	581	1.00	169	1.08 (0.90, 1.29)	
Yes	49	1.00	17	0.94 (0.53, 1.67)	

Abbreviations: HR indicates hazard ratio; CI, confidence interval; and MET, metabolic equivalent of task, BMI, body mass index; WHR, waist-to-hip ratio.

Multivariable model was adjusted for the same set of covariates of Supplementary table 2, as appropriate.

Meng R, *et al. Heart* 2020; 106:213–220. doi: 10.1136/heartjnl-2019-315750

Supplementary table 4. HRs (95% CIs) for association between early famine exposure and cancer according to potential baseline risk factors

	**Non-famine births**		**Famine births**		**$P_{interaction}$**
	**Cases**	**HR**	**Cases**	**HR (95% CIs)**	
**Daily smoking**					0.879
No	1,695	1.00	474	0.98 (0.88, 1.09)	
Yes	736	1.00	206	1.09 (0.93, 1.28)	
**Daily alcohol consumption**					0.741
No	2,136	1.00	601	1.00 (0.91, 1.09)	
Yes	295	1.00	79	1.13 (0.86, 1.47)	
**Physical activity, MET-hour/day**					0.229
<22.7	1,318	1.00	347	0.95 (0.84, 1.07)	
≥22.7	1,113	1.00	333	1.08 (0.95, 1.23)	
**BMI, kg/m^2**					0.304
<24.0	1,274	1.00	363	1.06 (0.93, 1.19)	
≥24.0	1,157	1.00	317	0.96 (0.84, 1.09)	
**WHR**					0.246
Men <0.90, women <0.85	1,091	1.00	297	0.96 (0.84, 1.10)	
Men ≥0.90, women ≥0.85	1,340	1.00	383	1.05 (0.93, 1.18)	
**Hypertension**					0.940
No	1,788	1.00	509	1.00 (0.90, 1.11)	
Yes	643	1.00	171	1.03 (0.87, 1.23)	
**Diabetes**					0.010
No	2,304	1.00	653	1.04 (0.95, 1.13)	
Yes	127	1.00	27	0.57 (0.37, 0.88)	

Abbreviations: HR indicates hazard ratio; CI, confidence interval; and MET, metabolic

Meng R, *et al. Heart* 2020; 106:213–220. doi: 10.1136/heartjnl-2019-315750

equivalent of task, BMI, body mass index; WHR, waist-to-hip ratio.

Multivariable model was adjusted for the same set of covariates of Supplementary table 2, as appropriate.

Supplementary table 5. HRs (95% CIs) for association between famine exposure and incident respiratory system disease according to potential baseline risk factors

	Non-famine births		Famine births		$P_{interaction}$
	**Cases**	**HR**	**Cases**	**HR (95% CIs)**	
**Respiratory system disease**					
**Daily smoking**					0.219
No	8,606	1.00	2,425	0.96 (0.92, 1.01)	
Yes	3,209	1.00	965	0.98 (0.91, 1.06)	
**Daily alcohol consumption**					0.046
No	10,859	1.00	3,115	0.96 (0.92, 1.00)	
Yes	956	1.00	275	1.04 (0.91, 1.20)	
**Physical activity, MET-hour/day**					0.593
<22.7	4,552	1.00	1,225	1.00 (0.93, 1.06)	
≥22.7	7,263	1.00	2,165	0.95 (0.91, 1.00)	
**BMI, kg/m^2**					0.116
<24.0	6,793	1.00	1,957	0.98 (0.93, 1.04)	
≥24.0	5,022	1.00	1,433	0.94 (0.89, 1.00)	
**WHR**					0.948
Men <0.90, women <0.85	5,347	1.00	1,551	0.96 (0.90, 1.02)	
Men ≥0.90, women ≥0.85	6,468	1.00	1,839	0.97 (0.92, 1.03)	
**Hypertension**					0.298
No	8,493	1.00	2,429	0.98 (0.93, 1.03)	
Yes	3,322	1.00	961	0.94 (0.88, 1.02)	
**Diabetes**					0.492
No	11,383	1.00	3,248	0.97 (0.93, 1.01)	
Yes	432	1.00	142	0.98 (0.80, 1.20)	

	Non-famine births		Famine births		$P_{interaction}$
	**Cases**	**HR**	**Cases**	**HR (95% CIs)**	
**COPD**					
**Daily smoking**					0.594
No	449	1.00	110	1.04 (0.83, 1.29)	
Yes	242	1.00	66	1.14 (0.86, 1.52)	
**Daily alcohol consumption**					0.852
No	617	1.00	161	1.06 (0.88, 1.27)	
Yes	74	1.00	15	1.02 (0.57, 1.84)	
**Physical activity, MET-hour/day**					0.573
<22.7	346	1.00	88	1.12 (0.88, 1.44)	
≥22.7	345	1.00	88	1.00 (0.79, 1.28)	
**BMI, kg/m^2**					0.407
<24.0	420	1.00	109	1.11 (0.89, 1.39)	
≥24.0	271	1.00	67	1.01 (0.76, 1.34)	
**WHR**					0.423
Men <0.90, women <0.85	284	1.00	79	1.17 (0.90, 1.52)	
Men ≥0.90, women ≥0.85	407	1.00	97	1.00 (0.79, 1.26)	
**Hypertension**					0.653
No	518	1.00	135	1.08 (0.89, 1.32)	
Yes	173	1.00	41	1.02 (0.71, 1.45)	
**Diabetes**					0.818
No	669	1.00	170	1.06 (0.89, 1.26)	
Yes	22	1.00	6	1.34 (0.50, 3.64)	

Abbreviations: HR indicates hazard ratio; CI, confidence interval; COPD, chronic obstructive pulmonary disease; and MET, metabolic equivalent of task, BMI, body mass index; WHR,

waist-to-hip ratio.

Multivariable model was adjusted for the same set of covariates of Supplementary table 2 except for family history, as appropriate.

Meng R, *et al. Heart* 2020; 106:213–220. doi: 10.1136/heartjnl-2019-315750
